# Predictors of pain improvement with Kampo formulas after UPOINT-guided management in chronic pelvic pain syndrome: a retrospective cohort study

**DOI:** 10.3389/fphar.2026.1859612

**Published:** 2026-08-27

**Authors:** Hiroyuki Kitano, Keiko Ogawa-Ochiai, Rei Ueno, Mai Okazaki, Kazuma Yukihiro, Tomoya Hatayama, Hiroyuki Shikuma, Yoshinori Nakano, Shinsaku Tasaka, Kyosuke Iwane, Ryo Tasaka, Yuki Kohada, Shunsuke Miyamoto, Miki Naito, Kohei Kobatake, Yohei Sekino, Keisuke Goto, Keisuke Hieda, Nobuyuki Hinata

**Affiliations:** 1 Department of Urology, Graduate School of Biomedical and Health Sciences, Hiroshima University, Hiroshima, Japan; 2 Kampo Clinical Center, Hiroshima University Hospital, Hiroshima, Japan

**Keywords:** chronic pelvic pain syndrome, kampo formulas, pain, predictive factors, UPOINT system

## Abstract

**Objectives:**

Chronic prostatitis or chronic pelvic pain syndrome is a heterogeneous condition commonly managed using the symptom-based Urinary, Psychosocial, Organ-specific, Infection, Neurologic/Systemic, and Tenderness (UPOINT) system; however, pain often persists despite targeted treatment. This study evaluated the effectiveness of traditional Japanese Kampo formulas as an additional therapy and sought to identify the clinical predictors of pain improvement in affected patients.

**Methods:**

We retrospectively analyzed 71 patients with chronic prostatitis or chronic pelvic pain syndrome who were treated according to the UPOINT system at a university hospital between May 2021 and January 2025. Among them, 37 received Kampo formulas in addition to standard therapy, while 34 received standard therapy alone. Outcomes were assessed using validated symptom questionnaires measuring prostatitis, urinary, and overactive bladder symptoms, and general health status. Changes in scores were compared between the groups, and multivariable logistic regression analysis was performed to identify the independent predictors of pain improvement associated with Kampo treatment.

**Results:**

Patients who repeatedly received Kampo formulas experienced penile or scrotal pain, and prostatic tenderness was elicited on examination; however, urinary symptoms were relatively mild. The Kampo group demonstrated a significantly greater improvement in pain scores than the standard therapy group, whereas improvements in urinary symptoms and quality of life were similar between the groups. Prostatic tenderness and pain-dominant symptoms were independently associated with pain improvement. No severe treatment-related adverse events were observed.

**Conclusion:**

Kampo formulas may provide effective additional pain relief for selected patients, particularly those with prostatic tenderness and predominant pain symptoms, thereby supporting more individualized treatment strategies. However, these findings should be interpreted with caution because of the retrospective study design, small sample size, baseline imbalances between the groups, and potential residual confounding related to the non-randomized selection of Kampo treatment. Prospective randomized studies are warranted to confirm these findings.

## 1 Introduction

Chronic prostatitis/chronic pelvic pain syndrome (CP/CPPS), classified as National Institutes of Health (NIH) category III, is a common male genitourinary condition characterized by chronic genitourinary pain in the absence of identifiable uropathogenic bacteria on standard microbiological evaluation (Krieger et al., 1999).

Despite its high prevalence and clinical significance, the etiology and underlying pathophysiology of CP/CPPS remain unclear, and no disease-modifying therapies have been established (Wenninger et al., 1996; Werneburg et al., 2021; Clemens et al., 2019). To address the heterogeneous nature of CP/CPPS, a phenotype-directed multimodal treatment strategy based on the Urinary, Psychosocial, Organ-specific, Infection, Neurologic/Systemic, and Tenderness) UPOINT (system has been proposed. This system categorizes patient symptoms into six domains: urinary, psychosocial, organ-specific, infectious, neurological/systemic, and skeletal muscle tenderness, and combines domain-specific therapies accordingly. Previous studies have demonstrated that UPOINT-based management can improve the overall symptoms in patients with CP/CPPS (Shoskes et al., 2009). However, pain often persists despite optimized UPOINT-directed therapy, which represents a critical therapeutic gap. Although antibiotics, nonsteroidal anti-inflammatory drugs, antidepressants, and anxiolytics are commonly prescribed for CP/CPPS, their clinical effectiveness is limited by several factors, including discordance between treatment and individual symptom profiles, incomplete resolution of residual symptoms, drug tolerance or resistance, and suboptimal long-term outcomes (Trofimenko et al., 2017). Therefore, adjunctive therapeutic options, tailored to specific symptom phenotypes, are required. In recent years, blood stasis syndrome was recognized as one of the major pathophysiological patterns in prostate diseases, and Chinese clinical practice guidelines recommend traditional Chinese patent medicines corresponding to Kampo formulas in Japan for its management (Medicine, 2022; Medicine, 2023).

Kampo formula extracts have been widely used in urological practice in Japan. One hundred forty-eight types of medical-use Kampo extract preparations are covered by health insurance in Japan and are used with established safety; however, evidence regarding their effectiveness and appropriate patient selection in CP/CPPS remains limited. Kampo medicine is characterized by individualized treatment based on pattern differentiation (“Sho”), whereby physicians select herbal formulas according to each patient’s symptoms and constitutional characteristics rather than using a single standardized treatment for a specific disease. In the present study, Kampo treatment consisted of insurance-approved extract formulations prescribed according to the patient’s Kampo pattern, with the most commonly used formulas including Keishibukuryogan, Choreito, Ryutanshakanto, and Hochuekkito.

This study aimed to evaluate the therapeutic efficacy of Kampo formulas as an adjunctive treatment in patients with CP/CPPS managed according to the UPOINT classification system, and to identify clinical factors associated with pain improvement.

## 2 Methods

### 2.1 Study design and participants

This retrospective observational study was approved by the Institutional Review Board of Hiroshima University (approval number: E2021-2,509). Men presenting at Hiroshima University Hospital with symptoms suggestive of CP/CPPS persisting for at least 6 months were eligible for inclusion.

Patients were excluded if they met the diagnostic criteria for NIH category I, II, or IV prostatitis syndrome; had received antibiotic treatment within the preceding 4 weeks; were currently using prophylactic antibiotics; or had an indwelling transurethral or suprapubic catheter. CP/CPPS was diagnosed according to the NIH category III criteria.

### 2.2 Treatment strategy based on the UPOINT system

All patients initially received phenotype-directed multimodal therapy according to the UPOINT classification system. Domain-specific treatment was provided based on the presence of positive UPOINT domains following our institutional treatment protocol. Patients with the urinary domain received α1-blockers and/or phosphodiesterase type 5 inhibitors. Patients with the psychosocial domain received patient education and supportive counseling during outpatient visits; formal cognitive-behavioral therapy or psychologist-led interventions were not routinely performed. Patients with the organ-specific domain received nonsteroidal anti-inflammatory drugs or acetaminophen. Patients with documented bacterial infection received pathogen-directed antimicrobial therapy. Patients with neurologic/systemic symptoms received neuropathic pain medications, including pregabalin, when appropriate. Patients with tenderness of the pelvic floor or skeletal muscles underwent stretching exercises and were referred for acupuncture therapy. After at least approximately 3 months of UPOINT-guided management, treatment strategies were re-evaluated according to changes in clinical symptoms. Because some patients had received previous treatment at referring institutions before presentation to our hospital, the duration of UPOINT-guided management varied among individuals. Patients whose symptoms remained manageable continued UPOINT-guided therapy alone (control group). In contrast, patients with persistent symptoms, particularly pain-dominant symptoms despite ongoing UPOINT-guided management, were considered for adjunctive Kampo treatment based on the attending physician’s clinical judgment and the patient’s preference (Kampo group). The Kampo formulas used in this study included Keishibukuryogan, Choreito, Ryutanshakanto, and Hochuekkito, all of which are prescription Kampo extract formulations approved and covered by the Japanese national health insurance system. The specific Kampo formula was selected according to symptom severity, clinical symptoms, physical findings, Kampo diagnosis (Sho), disease characteristics, and the patient’s preference, reflecting routine clinical practice in Japan. In selected cases, treatment selection was made in consultation with a physician experienced in Kampo medicine. The aim of this study was to determine whether the addition of Kampo formulas to ongoing UPOINT-guided management provided additional clinical benefit compared with continued UPOINT-guided management alone.

### 2.3 Outcome measures

Treatment outcomes were assessed using validated patient-reported outcome measures, including the NIH Chronic Prostatitis Symptom Index (NIH-CPSI), International Prostate Symptom Score (IPSS), and Overactive Bladder Symptom Score (OABSS). Additionally, response to the following item from the Geriatric 8 (G8) screening tool was obtained: “In comparison with other people of the same age, how does the patient consider their health status?” (Blanchard et al., 1993). These assessments were performed at baseline and at follow-up.

The primary outcome was symptom improvement, which was defined as a reduction in the NIH-CPSI score during the observation period. Secondary outcomes included changes in IPSS, OABSS, and health-related indices such as the G8 score.

### 2.4 Clinical variables

Baseline clinical data were extracted from medical records, including age, body mass index (BMI), symptom duration, pain location, prostate volume (PV), presence of prostatic tenderness on digital rectal examination, baseline symptom scores, and UPOINT domain classification.

### 2.5 Statistical analysis

Continuous variables were presented as median values with interquartile ranges or mean values with standard deviations, as appropriate. Categorical variables were expressed as frequencies and percentages. Comparisons between groups were performed using the Mann–Whitney U test for continuous variables and the chi-square test or Fisher’s exact test for categorical variables. Univariable logistic regression analysis was first performed to identify potential clinical predictors of pain improvement associated with Kampo therapy. Variables with a *P* value < 0.1 in the univariable analysis were subsequently included in the multivariable logistic regression model to identify independent predictors. The variables included in the multivariable model (body mass index, prostate volume, and prostatic tenderness) were considered clinically relevant factors reflecting disease severity and treatment responsiveness in patients with CP/CPPS. Statistical significance was defined as a two-sided *P* value < 0.05. All statistical analyses were performed using JMP Pro version 18.1.0 (SAS Institute Inc., Cary, NC, United States of America).

## 3 Results

### 3.1 Patient background

A total of 71 Japanese men were included in this study. None of the patients had pyuria or bacteriuria in their pre- or post-massage urine samples and were diagnosed with category IIIb prostatitis (CP/CPPS category IIIb). Their symptoms were classified according to the UPOINT system; treatment consisted of an α1-blocker or a phosphodiesterase type 5 inhibitor for urinary symptoms; Cernilton, Eviprostat, or acetaminophen for organ-specific symptoms; and pregabalin for skeletal muscle tenderness. After treatment based on the UPOINT framework, 34 patients requested that Kampo formulas be added to their treatment regimen. Table 1 summarizes the clinical characteristics of the patients who received Kampo formulas (Kampo-treated group) and those who did not (non-Kampo-treated group). No significant differences were observed between the two groups in terms of age, BMI, number of previous treatments, Prostate Volume, uroflowmetry parameters (Qmax, average flow rate, voided volume, and residual volume), NIH-CPSI scores for urinary symptoms, or quality of life (QOL). However, patients in the Kampo group more frequently reported pain at the penile tip and exhibited prostatic tenderness on examination. The NIH-CPSI total and pain domain scores were significantly higher in this group, and urinary symptoms were significantly worse than those in the non-Kampo-treated group.

**TABLE 1:** Baseline characteristics of patients according to Kampo medicine use.

Variable	UPOINT + Kampo (n = 37)	UPOINT alone (n = 34)	p value
Demographic characteristics			
Age, years	52.6 (19.2)	50.7 (17.5)	0.68
Body mass index, kg/m²	23.1 (3.4)	22.8 (3.1)	0.63
Number of prior medications	2.9 (1.8)	2.7 (1.8)	0.58
Smoking status (None : Former : Current)	16 : 11 : 7	24 : 7 : 3	0.13
Prostate-related parameters			
Prostate volume, mL	26.6 (10.4)	26.7 (16.4)	0.96
Prostatic tenderness, n (%)	18 (48.6)	7 (18.9)	0.005
Pain characteristics			
Pain location	​	​	0.004
–Area between anus and scrotum	13	14	​
–Testicles or scrotum, n (%)	4	3	​
–Tip of the penis	13	3	​
–Below the waist (pubic/bladder area), n (%)	4	14	​
NIH-CPSI total score	25.8 (4.2)	22.4 (7.1)	0.021
NIH-CPSI pain domain score	13.8 (2.9)	11.2 (3.7)	0.002
NIH-CPSI urinary domain score	3.1 (2.4)	3.4 (3.0)	0.68
NIH-CPSI QOL impact score	8.7 (2.2)	8.5 (1.8)	0.75
Uroflowmetry parameters			
Maximum flow rate (Qmax), mL/s	21.6 (11.2)	18.0 (9.1)	0.17
Average flow rate, mL/s	14.1 (6.8)	11.8 (6.1)	0.17
Voided volume, mL	322.8 (193.9)	243.5 (144.6)	0.08
Post-void residual volume, mL	25.0 (19.3)	29.2 (36.3)	0.57
Other symptom scores			
IPSS total score	6.6 (6.2)	12.3 (9.8)	0.0005
IPSS quality-of-life score	3.3 (2.3)	4.0 (1.8)	0.23
OABSS	3.0 (2.6)	3.2 (2.6)	0.78
G8 health status score	0.52 (0.64)	0.47 (0.62)	0.76

### 3.2 Kampo formulas

Among the Kampo formulas prescribed, Keishibukuryogan was the most frequently used (n = 29), followed by Choreito (n = 4), Hochuekkito (n = 2), and Ryutanshakanto (n = 2). No other Kampo formula was prescribed in more than one patient.

### 3.3 Effectiveness

Two-way repeated-measures analysis of variance (ANOVA) with type III sums of squares was performed to evaluate the changes in the NIH-CPSI total and domain scores over time between the Kampo-treated and non-Kampo-treated groups.

### 3.4 NIH-CPSI total scores

**FIGURE 1:**
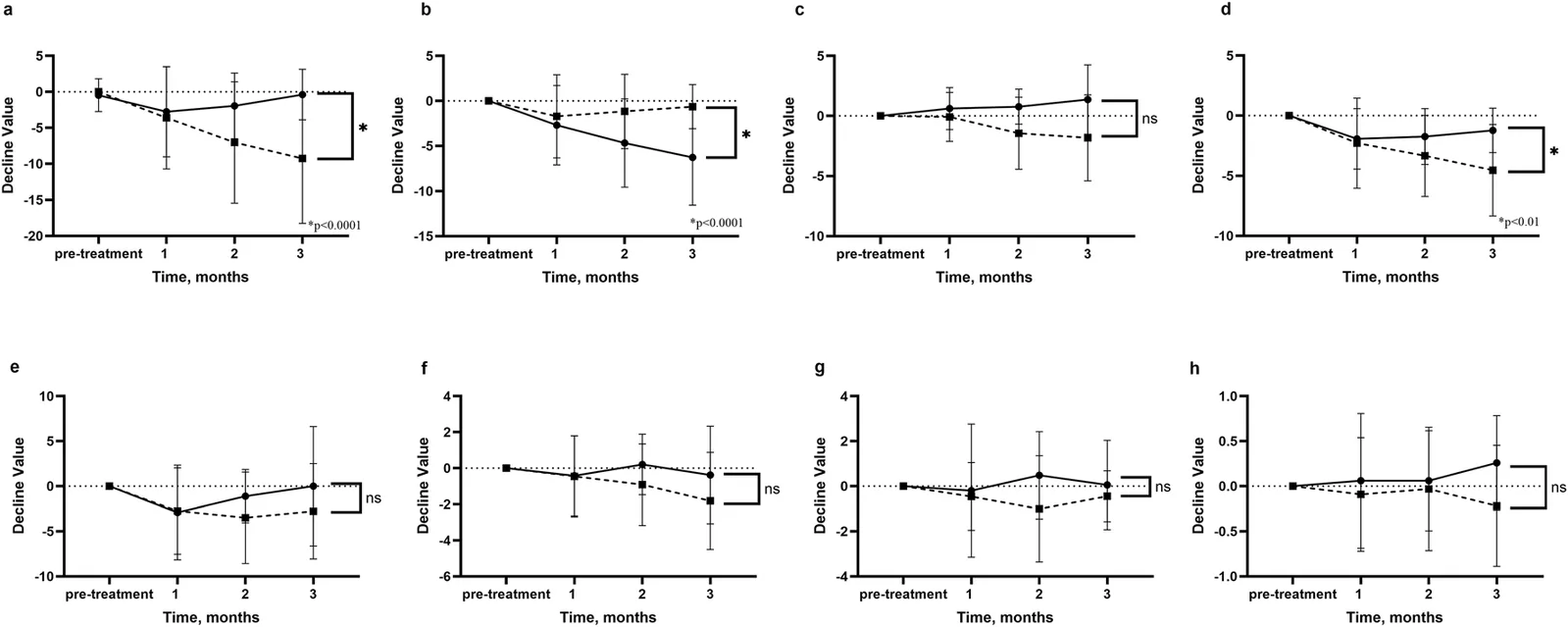
Time-course changes in symptom scores following treatment in patients with chronic prostatitis/chronic pelvic pain syndrome (CP/CPPS) category IIIb Time-dependent changes in symptom scores over a 3-month period were compared between patients treated with Urinary, Psychosocial, Organ-specific, Infection, Neurologic/systemic, and Tenderness (UPOINT)-based therapy alone and those treated with additional Kampo medicine. **(a)** Total National Institutes of Health Chronic Prostatitis Symptom Index (NIH-CPSI) score. **(b)** NIH-CPSI pain domain score. **(c)** NIH-CPSI urinary symptom score. **(d)** NIH-CPSI quality-of-life (QOL) impact domain score. **(e)** International Prostate Symptom Score (IPSS). **(f)** IPSS quality-of-life score. **(g)** Overactive Bladder Symptom Score (OABSS). **(h)** Geriatric 8 (G8) health status score. The solid line (●) represents the UPOINT + Kampo group, and the dashed line (■) represents the UPOINT-alone group. A significantly greater reduction in the NIH-CPSI pain domain score was observed in the Kampo group, whereas improvements in urinary symptoms were mainly attributable to UPOINT-based therapy.

The total NIH-CPSI scores differed significantly between the groups (P < 0.0001), with greater improvement observed in the non-Kampo-treated group. Significant effects of time and group-by-time interaction was observed (both P < 0.0001) (Figure 1a).

### 3.5 NIH-CPSI pain domain scores

The NIH-CPSI pain scores differed significantly between the groups, with greater improvement observed in the Kampo-treated group. A two-way ANOVA revealed significant effects of group, time, and group-by-time interaction (P < 0.0001, P < 0.0001, and P = 0.0011, respectively) (Figure 1b).

### 3.6 NIH-CPSI urinary symptom scores

The NIH-CPSI urinary symptom scores showed a significant effect of time and a significant group-by-time interaction (P < 0.0001 and P = 0.0004, respectively), whereas no significant overall group effect was observed (P = 0.4043) (Figure 1c).

### 3.7 NIH-CPSI impact domain scores

The NIH-CPSI QOL scores differed significantly between the groups, with greater improvements observed in the non-Kampo-treated group. Significant effects of time and group-by-time interaction was also observed (P = 0.0002 and P = 0.0087, respectively) (Figure 1d).

### 3.8 IPSS scores

The IPSS differed significantly between the groups, with greater improvement observed in the non-Kampo-treated group (P = 0.0011). A significant effect of time was observed (P = 0.0462); however, no significant group-by-time interaction was observed (P = 0.1926) (Figure 1e).

### 3.9 IPSS QOL scores

The IPSS-QOL scores showed a significant effect of time (P = 0.0161), whereas no significant group effect (P = 0.0503) or group-by-time interaction (P = 0.1355) was observed (Figure 1f).

### 3.10 OABSS

The OABSS demonstrated a modest effect of time (P = 0.0449), with no significant group effect (P = 0.7785) or group-by-time interaction (P = 0.1525) (Figure 1g).

### 3.11 G-8 health status domain

The G8 health status scores showed a modest effect of time (P = 0.0113), whereas no significant group effect (P = 0.9841) or group-by-time interaction (P = 0.1258) was observed (Figure 1h).

### 3.12 Predictors of pain improvement according to baseline clinical characteristics

#### 3.12.1 Age

**FIGURE 2:**
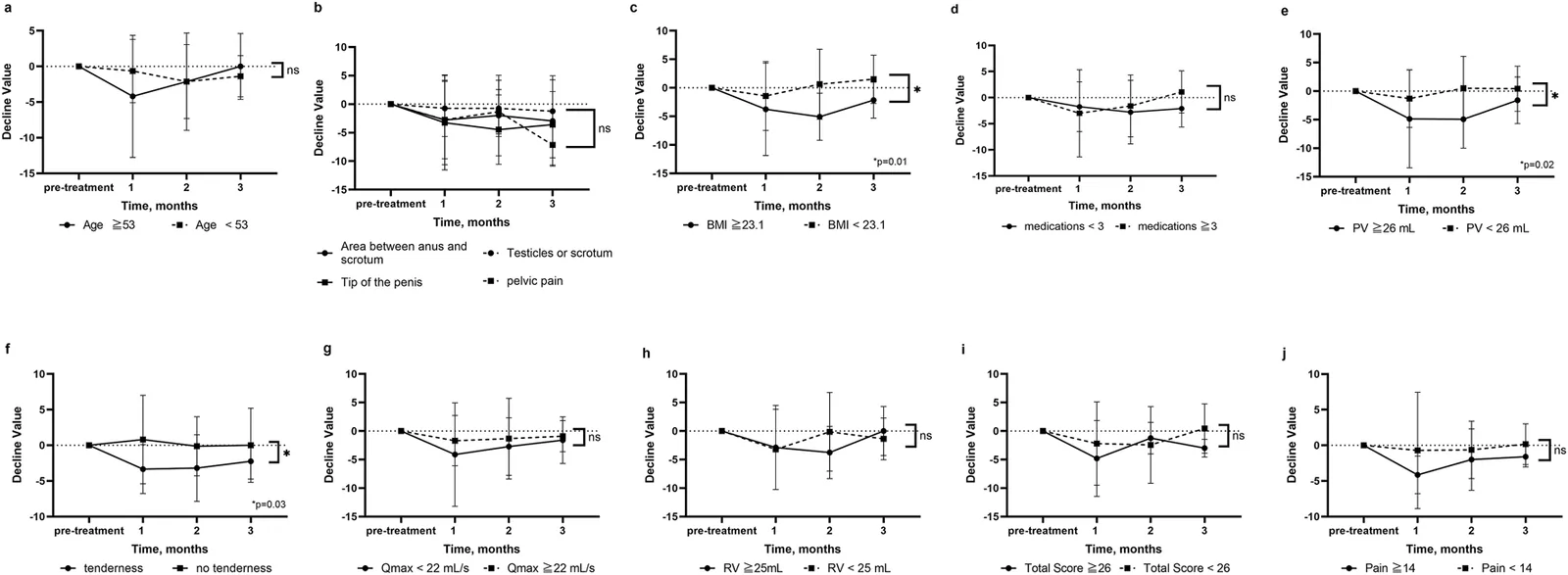
Subgroup analyses of time-course changes in the NIH-CPSI pain domain score following Kampo treatment. Time-dependent changes in the NIH-CPSI pain domain score over a 3-month period were evaluated in the Kampo group after stratification according to baseline clinical characteristics. The patients were divided into two groups based on the mean value of each variable used as the cutoff. **(a)** Age (≥ mean vs. < mean). **(b)** Pain location (testicles/scrotum vs. other sites) **(c)** Body mass index (BMI ≥ mean vs. < mean). **(d)** Number of prior treatments (≥ mean vs. < mean). **(e)** Prostate volume (≥ mean vs. < mean). **(f)** Prostatic tenderness (present vs. absent). **(g)** Maximum flow rate (Qmax ≥ mean vs. < mean). **(h)** Post-void residual volume (≥ mean vs. < mean). **(i)** NIH-CPSI total score (≥ mean vs. < mean). **(j)** NIH-CPSI pain domain score (≥ 14 vs. < 14).

No significant difference in pain improvement was observed between the age groups based on the mean age value (Figure 2a).

#### 3.12.2 Pain location

Pain improvement differed significantly according to the pain location (P = 0.0070), whereas no significant change over time was observed (P = 0.7528) (Figure 2b).

#### 3.12.3 BMI

Pain improvement differed significantly according to BMI category, with greater reductions observed in patients with BMI ≥ 23.1. Significant effects of BMI, time, and their interaction was observed (P = 0.0119, P = 0.0001, and P = 0.0150, respectively) (Figure 2c).

#### 3.12.4 Prior treatment

Pain improvement differed modestly according to the number of previous treatments (P = 0.0367), whereas no significant effect of time (P = 0.3217) or group-by-time interaction (P = 0.3140) was observed (Figure 2d).

#### 3.12.5 Prostate volume

Pain improvement differed significantly according to PV, with greater reductions observed in patients with PV ≥ 26 mL. Significant effects of PV group, time, and their interaction were observed (P = 0.0039, P = 0.0003, and P = 0.0214, respectively) (Figure 2e)

#### 3.12.6 Pain tenderness

Pain improvement did not differ significantly according to the presence of prostatic tenderness (P = 0.2721), and no significant interaction effect was observed (P = 0.1137). However, a significant effect of time was observed (P = 0.0004) (Figure 2f).

#### 3.12.7 Max flow rate

Pain improvement differed modestly according to the baseline Qmax (P = 0.0196), whereas no significant effect of time (P = 0.1670) or group-by-time interaction (P = 0.6498) was observed (Figure 2g).

#### 3.12.8 Residual urine volume

Pain improvement differed modestly according to the baseline residual urine volume (P = 0.0108), whereas no significant effect of time (P = 0.5460) or group-by-time interaction (P = 0.1582) was observed (Figure 2h).

#### 3.12.9 NIH-CPSI total score

Pain improvement differed modestly according to the baseline NIH-CPSI total score (P = 0.0062), whereas no significant effect of time (P = 0.1581) or group-by-time interaction (P = 0.2207) was observed (Figure 2i).

#### 3.12.10 NIH-CPSI pain domain score

Pain improvement differed according to the baseline NIH-CPSI pain score (P = 0.0152), with greater reductions observed in patients with higher baseline pain severity (≥ 14). A significant time effect was observed (P = 0.0093), but no significant group-by-time interaction was observed (P = 0.1563) (Figure 2j).

#### 3.12.11 Univariable and multivariable logistic regression analyses of factors associated with treatment response

In the univariable analysis, PV was significantly associated with treatment response (odds ratio (OR) 1.20 per 1 mL increase, 95% confidence interval (CI) 1.04–1.48, P = 0.038), and prostate tenderness showed a trend toward significance (OR 5.25, 95% CI 0.77–49.9, P = 0.09). BMI was not significantly associated with the outcome (P = 0.75).

Given the small sample size, the number of variables included in the multivariable model was limited to reduce the risk of overfitting. Therefore, only three clinically relevant variables (BMI, PV, and prostate tenderness) were included in the final multivariable logistic regression model (Table 2).

**TABLE 2:** Univariable and multivariable logistic regression analyses of factors associated with treatment response.

Variable	Univariate OR (95% CI)	Univariate p value	Multivariate OR (95% CI)	Multivariate p value
Body mass index	0.95 (0.72-1.27)	0.75	0.85 (0.55-1.23)	0.40
Prostate volume	1.20 (1.04-1.48)	0.038	1.23 (1.06-1.59)	0.037
Tenderness of prostate				
No	Reference	0.09	Reference	0.0026
Yes	5.25 (0.77-49.9)	​	19.27 (1.17-1564.5)	​

In the multivariable analysis, PV remained independently associated with treatment response (OR 1.23 per 1 mL increase, 95% CI 1.06–1.59, P = 0.037). The presence of prostate tenderness (reference: absent) was also independently associated with treatment response (OR 19.27, 95% CI 1.17–1564.5, P = 0.037), whereas BMI was not significantly associated with the outcome (OR 0.85, 95% CI 0.55–1.23, P = 0.40).

## 4 Discussion

In this study, we evaluated the therapeutic effects of Kampo formulas on CP/CPPS, in patients managed according to the UPOINT system. Compared with patients who received UPOINT-based management alone, those who received adjunctive Kampo therapy demonstrated a significantly greater reduction in the NIH-CPSI pain domain score. In contrast, the improvements in urinary symptoms and QOL scores were comparable between the two groups.

Furthermore, multivariable analysis revealed that pain improvement following Kampo treatment was independently associated with a larger PV and the presence of prostatic tenderness. These findings suggest that Kampo formulas may be particularly beneficial for specific CP/CPPS phenotypes characterized by organ-specific features and pain-dominant symptoms.

Community-based surveys have revealed a CPPS prevalence of approximately 8% (Nickel et al., 2003), with some studies reporting rates as high as 11.5% among men younger than 50 years (Suskind et al., 2013). These data show that CPPS is relatively common. Previous reports have proposed several potential etiologies of chronic prostatitis, including bacterial prostatitis (Kitano et al., 2025), lower urinary tract dysfunction, autoimmune mechanisms, and pelvic floor muscle spasm (Shoskes, 2001).

However, the precise underlying cause remains unclear. Therefore, current treatment strategies primarily target individual symptom domains rather than providing a definitive disease-modifying approach (Shoskes et al., 2009).

Kampo formulas, a traditional Japanese medical system derived from Traditional Chinese Medicine, emphasize individualized treatment based on pattern differentiation. When the underlying pattern is consistent, the same herbal formula can be used to treat different diseases. In the context of prostate diseases, blood stasis syndrome is implicated in prostate disease onset and progression (Wang et al., 2025). In a study involving 400 patients with prostatitis, 36.5% of the patients had blood stasis syndrome (Kong, 2020). Similarly, among 200 patients with benign prostatic hyperplasia, 27% were diagnosed with blood stasis syndrome, which was positively correlated with the IPSS (Huang et al., 2014). Furthermore, the latest Chinese clinical guidelines recognize blood stasis syndrome as a major syndrome pattern in prostate diseases and recommend herbal formulas such as Guizhi Fuling Wan (Wang et al., 2025; The Andrology Division of the China Association of Chinese Medicine Informatics, 2022). Kampo formulas consist of multiple herbal components and have been shown in basic research to exert anti-inflammatory effects. These effects include suppression of pro-inflammatory cytokines, such as interleukin-1β and tumor necrosis factor-α, as well as inhibition of the inflammatory transcription factor nuclear factor kappa B and inflammasome components (NLRP3, caspase-1, and ASC) (Zang et al., 2021). In addition, Kampo formulations may reduce nitric oxide production by modulating muscarinic acetylcholine receptors, thereby contributing to their anti-inflammatory activity (Zhou et al., 2024).

The complex glandular architecture of the prostate complicates the treatment of CP/CPPS (Oliveira et al., 2016). Once inflammation develops, tissue edema can obstruct the glandular ducts, thereby impairing the penetration of therapeutic agents into the prostatic tissues and reducing their ability to eradicate pathogens (Fitzgerald et al., 2013). Furthermore, obstruction of the prostatic ducts hinders the drainage of inflammatory substances, which may contribute to persistent or recurrent inflammation (Yavaşçaoğlu et al., 1999). Based on these pathophysiological considerations, anti-inflammatory agents represent a rational therapeutic approach for chronic prostatitis. Therefore, Kampo formulations with anti-inflammatory properties might have clinical benefits.

The relatively favorable effect on pain observed in this study may, at least in part, be attributable to their anti-inflammatory actions.

Although administered in combination with prostatic massage, treatment with Guizhi Fuling Wan in patients with CPPS resulted in a significant reduction in the NIH-CPSI total score, from 10.10 ± 1.77 to 3.00 ± 1.75. Perineal pain assessed using the visual analog scale, decreased significantly from 5.65 ± 3.25 to 1.60 ± 0.68. Furthermore, 85% of patients achieved a symptom improvement rate of ≥ 60%, indicating a substantial clinical response (Wu et al., 2024). In the present study, although Guizhi Fuling Wan was the most frequently prescribed Kampo formula, several other formulas were also prescribed according to each patient’s Kampo diagnosis (Sho) and clinical presentation. Guizhi Fuling Wan is generally indicated for patients with blood stasis syndrome, whereas other Kampo formulas are selected for different Kampo diagnoses and symptom profiles. Therefore, the therapeutic effects observed in the present study likely reflect the individualized nature of Kampo medicine rather than the efficacy of a single Kampo formula. This heterogeneity may have influenced the magnitude of the observed treatment effects and should be taken into consideration when interpreting the study findings.

Pain is a central and distressing symptom of CP/CPPS. CP/CPPS-associated pain is most commonly localized to the pelvis, perineum, and genital region, and is frequently accompanied by lower urinary tract symptoms and sexual dysfunction. Collectively, these symptoms can substantially impair patients’ QOL and are often associated with psychological distress, including depression and anxiety (Wenninger et al., 1996; Tripp et al., 2013). In this study, the addition of Kampo formulas after UPOINT-guided multimodal therapy was associated with further pain reduction in patients with refractory CP/CPPS (Wu et al., 2024). Although these findings should be interpreted with caution, Kampo formulas may represent a potential adjunctive therapeutic option for patients who do not adequately respond to conventional treatment strategies.

## 5 Limitations

This study had several limitations. First, the sample size was relatively small, which may have reduced the precision of the estimates and limited the generalizability of the findings. In particular, the wide confidence intervals observed for some predictors, such as prostatic tenderness, indicate that these results should be interpreted with caution and regarded as exploratory. Second, because Kampo formulas were prescribed according to physicians’ clinical judgment and patients’ preferences, selection bias and confounding by indication may have influenced treatment allocation. Although multivariable analyses were performed to adjust for measured confounders, residual confounding cannot be excluded. Accordingly, the observed association between adjunctive Kampo therapy and pain improvement should be interpreted with caution, and causality cannot be established because of the retrospective observational design. Third, the Kampo treatment group included several different formulas prescribed according to each patient’s Kampo diagnosis (Sho), reflecting routine individualized Kampo practice. Although Keishibukuryogan was the most frequently prescribed formula, the small number of patients receiving the other Kampo formulas precluded separate analyses of individual formulations. Furthermore, because Kampo formulas are multi-herb preparations selected according to each patient’s Kampo pattern rather than a standardized disease-specific regimen, it was not possible to evaluate the therapeutic effects of individual herbal ingredients or to attribute the observed benefits to a specific pharmacological mechanism. Fourth, access to Kampo formulas varies across regions and countries, and their availability may be limited to specific healthcare settings, thereby restricting the external applicability of our findings. Therefore, larger prospective multicenter studies with more diverse populations and standardized Kampo treatment protocols are warranted to validate our findings.

## Data Availability

The raw data supporting the conclusions of this article will be made available by the authors, without undue reservation.
